# The crystal structure of alanine racemase from *Streptococcus pneumoniae*, a target for structure-based drug design

**DOI:** 10.1186/1471-2180-11-116

**Published:** 2011-05-25

**Authors:** Hookang Im, Miriam L Sharpe, Ulrich Strych, Milya Davlieva, Kurt L Krause

**Affiliations:** 1 Research Institute of Pharmaceutical Sciences, College of Pharmacy, Seoul National University, Seoul, Korea; 2 Department of Biochemistry, University of Otago, Dunedin, New Zealand; 3 Department of Biology and Biochemistry, University of Houston, Houston, TX, USA; 4 Department of Biochemistry and Cell Biology, Rice University, Houston, TX, USA

## Abstract

**Background:**

*Streptococcus pneumoniae *is a globally important pathogen. The Gram-positive diplococcus is a leading cause of pneumonia, otitis media, bacteremia, and meningitis, and antibiotic resistant strains have become increasingly common over recent years. Alanine racemase is a ubiquitous enzyme among bacteria and provides the essential cell wall precursor, D-alanine. Since it is absent in humans, this enzyme is an attractive target for the development of drugs against *S. pneumoniae *and other bacterial pathogens.

**Results:**

Here we report the crystal structure of alanine racemase from *S. pneumoniae *(Alr_SP_). Crystals diffracted to a resolution of 2.0 Å and belong to the space group P3_1_21 with the unit cell parameters a = b = 119.97 Å, c = 118.10 Å, α = β = 90° and γ = 120°. Structural comparisons show that Alr_SP _shares both an overall fold and key active site residues with other bacterial alanine racemases. The active site cavity is similar to other Gram positive alanine racemases, featuring a restricted but conserved entryway.

**Conclusions:**

We have solved the structure of Alr_SP_, an essential step towards the development of an accurate pharmacophore model of the enzyme, and an important contribution towards our on-going alanine racemase structure-based drug design project. We have identified three regions on the enzyme that could be targeted for inhibitor design, the active site, the dimer interface, and the active site entryway.

## Background

Despite great advances in the development of antibiotics, the most common cause of community-acquired pneumonia, *Streptococcus pneumoniae*, is still a globally important pathogen, especially in children and the elderly [1]. This Gram-positive diplococcus is a leading cause not only of pneumonia, but also otitis media, bacteremia, and meningitis [2,3]. In children, *S. pneumoniae *is estimated to cause more than one-third of the 2 million deaths due to acute respiratory infections [4,5]. In the elderly, *S. pneumoniae *is the most common cause of fatal community-acquired pneumonia [6,7]. In adults from industrialized countries, pneumococcal pneumonia accounts for at least 30% of all cases of community-acquired pneumonia admitted to hospital, with a fatality rate of 11% to 44% [4]. In addition, co-infection of influenza patients with *S. pneumoniae *is known to exacerbate their clinical outcome [4]: for example, 50% or more of the flu-associated mortality in the 1918-1919 Spanish Flu epidemic is believed to have resulted from pneumococcal superinfections [8,9], and *S. pneumoniae *co-infection has been specifically correlated with the severity of the recent H1N1 pandemic influenza [10]. The rate of antibiotic resistance in *S. pneumoniae *has escalated dramatically since penicillin-resistant strains were first detected in the 1970s [11-15]. About 40% of pneumococcal isolates displayed multidrug-resistant phenotypes (resistance to three or more antibiotics) across 38 countries in 2004 [16,17]. To meet the challenge of increasing pneumococcal drug resistance it will be important to isolate new therapeutic compounds effective against *S. pneumoniae *through the identification of new target enzymes and the development of effective inhibitors to these targets.

The bacterial enzyme alanine racemase (Alr; E.C. 5.1.1.1) uses a covalently-bound pyridoxal 5"-phosphate (PLP) cofactor to catalyze the racemization of L-alanine and D-alanine, the latter being an essential component of the peptidoglycan layer in bacterial cell walls [18]. Microbiological evidence has consistently shown this enzyme to be essential in bacteria [19]. One apparent exception was found for the *Mycobacterium smegmatis *enzyme, which was able tolerate an insertion in its alanine racemase gene [20]. But this exception was disproved with the report of an alanine racemase deletion mutant in *M. smegmatis *that did not grow without D-alanine supplementation [19]. *S. pneumoniae*, unlike *Escherichia coli *or *Pseudomonas aeruginosa*, contains only one gene that codes for alanine racemase [21]. The lack of alanine racemase function in eukaryotes [22] makes this enzyme an attractive target for antimicrobial drug development. Structural studies are crucial to structure-based drug design [23-25], and solving the crystal structure of alanine racemase from *S. pneumoniae *(Alr_SP_) is a crucial step towards designing inhibitors of this enzyme.

To date, crystal structures of alanine racemase enzymes from seven different bacteria have been published: *Geobacillus stearothermophilus *(Alr_GS_) [26-31], *P. aeruginosa *(DadX_PA_) [32], *Streptomyces lavendulae *(Alr_SL_) [33], *Mycobacterium tuberculosis *(Alr_MT_) [34], *Bacillus anthracis *(Alr_BA_) [35,36], *E. coli *(Alr_EC_) [37], and *Enterococcus faecalis *(Alr_EF_) [38]. Structures of this enzyme from a further six microorganisms have been deposited in the PDB: *Bartonella henselae *(PDB ID 3KW3), *Oenococcus oeni *(3HUR and 3CO8), *Pseudomonas fluorescens *(2ODO), *Actinobacillus succinogenes *(3C3K), *Corynebacterium glutamicum *(2DY3), and *Staphylococcus aureus *(3OO2). In all of these structures, Alr is a homodimeric enzyme formed by a head-to-tail association of two monomers. Each monomer is composed of an N-terminal α/β barrel and an extended β-strand domain at the C-terminus. The active site in each monomer is located in the centre of the α/β barrel and contains a pyridoxal phosphate (PLP) co-factor covalently connected to a lysine residue by an internal aldimine bond. The catalytic mechanism is thought to involve two bases, the same lysine, and a tyrosine contributed by the opposite monomer [30,39,40]. The entryway to the active site and the PLP binding site consists of residues from loops in the α/β barrel domain of one monomer and residues from the C-terminal domain of the other monomer, and is roughly conical, with its base oriented toward the outside of the enzyme [34]. Structures of alanine racemase in complex with substrate analogs [27,28,30-32] and site-directed mutagenesis of the enzyme [31,40,41] have elucidated the reaction mechanism of the enzyme and verified the key roles of active site residues. Structures of alanine racemase complexed with alanine phosphonate and D-cycloserine (DCS) show that these inhibitors covalently bind to the PLP cofactor, which explains their ability to inhibit eukaryotic PLP-containing enzymes in a non-specific manner [27,30,37,38].

Determining the structure of alanine racemase from a range of bacterial species is an important step towards its full characterization in anticipation of inhibitor design. Here, we report the structure of Alr_SP_, and compare it with other published alanine racemase structures. This crystal structure will contribute useful information towards our structure-based drug design research aimed at the identification and development of alanine racemase inhibitors.

## Results and discussion

### Structure determination and refinement

Crystals of Alr_SP _suitable for X-ray diffraction were grown as described previously [21]. Crystals diffracted to a resolution of 2.0 Å and belong to the space group P3_1_21 with the unit cell parameters a = b = 119.97 Å, c = 118.10 Å, α = β = 90° and γ = 120°. The structure of Alr_SP _was solved by molecular replacement using CNS [42] and Alr_GS _(PDB ID 1SFT) [29] without the PLP cofactor as a search model. Refinement was carried out initially with CNS, then completed with TLS refinement [43] in Refmac5 [44]. After structure solution and refinement, the final model of Alr_SP_, validated using PROCHECK [45] has 92.7% of residues in the most favored regions of the Ramachandran plot, 6.9% of residues in the additionally allowed regions and 0.3% of residues in the generously allowed regions. The structure has root-mean-square (r.m.s.) deviations from ideality for bond lengths of 0.015 Å and for angles of 1.45°. Further data collection and refinement statistics are presented in Table 1.

**Table 1:** Data collection and structure refinement statistics

Data collection	
Unit cell parameters	a = 119.97 Å, b = 119.97 Å, c = 118.10 Å
	α = 90°, β = 90°, γ = 120°
Space group	P3_1_21
λ (Å)	1.5418
Mosaicity	0.48
Observations	475265
Unique reflections	66748
R-merge^*a *^(%)	8.3 (68.2)
Completeness (%)	99.6 (95.4)
<I/σ>	21.3 (1.7)

Refinement statistics	

Resolution (Å)	23.03 - 2.00 (2.05 - 2.00)
Reflections	63336 (4412)
Total atoms	6161
R-factor^b ^(%)	16.8 (32.2)
R_free _(%)	20.0 (35.5)
Average B-factors (Å^2^)	
Wilson B-factor	33.2
All atoms	42.7
Main chain atoms	41.8
Side chain atoms and waters	43.6
Waters	44.5
R.m.s. deviations	
Bond lengths (Å)	0.015
Bond angles (deg)	1.45
No. of residues	734, 100%
No. of protein atoms	5615
No. of PLP atoms	30
No. of benzoic acid atoms No. of water molecules	9 507
	
Residues in the Ramachandran plot	
Most favored regions	588, 92.7%
Additionally allowed regions	44, 6.9%
Generously allowed regions	2, 0.3%
Disallowed regions	0, 0%
	

^*a *^R-merge = Σ|*I*_obs_-*I*_avg_|/Σ|*I*_avg_|

^b ^R-factor = Σ|*F*_obs_-*F*_calc_|/Σ|*F*_obs_|

Values in parenthesis are for the highest resolution shell.

### Overall structure of Alr_SP_

**Figure 1:**
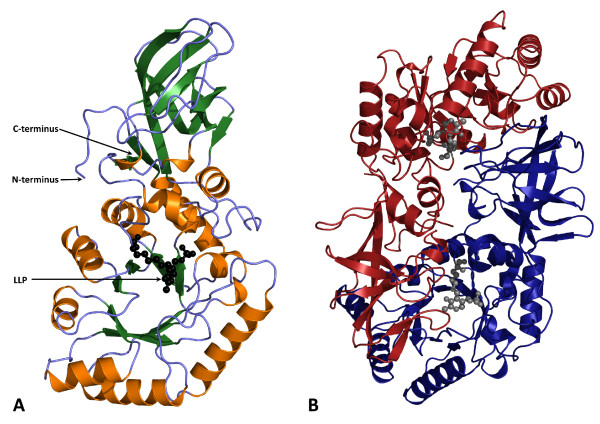
**Structure of alanine racemase from *S. pneumoniae***. (A) Ribbon diagram of the alanine racemase monomer with β-sheets colored green and α-helices colored gold. (B) Ribbon diagram of the alanine racemase dimer where one monomer is colored blue and the opposite monomer red. The N'-pyridoxyl-lysine-5'-monophosphate or LLP residue (PLP cofactor covalently bound to lysine; black or grey spheres) resides in the α/β barrel domain of the active site. The active site is composed of residues from the α/β barrel domain of one monomer and residues from the β-strand domain of the other monomer.

Alr_SP _forms a homodimer in which the two monomers form a head-to-tail association, typical of that seen in other alanine racemases. Each monomer has an eight-stranded α/β barrel domain (residues 1-238) and an extended β-strand domain (residues 239-367) (Figure 1A). The α/β barrel of one monomer is in contact with the β-strand domain of the other monomer (Figure 1B). Although the two monomers have very similar folds, they are crystallographically distinct in this crystal form (Table 1), and noncrystallographic symmetry was not used in refinement. The r.m.s. difference between the Cα atoms of the two monomers after superposition is 0.38 Å, and the average B-factors of monomers A and B are 38.4 and 46.9 Å^2^, respectively. As with other alanine racemases, the Alr_SP _homodimer contains two active sites, each composed of residues from the α/β barrel of one monomer and residues from the β-strand domain of the other. The pyridoxal phosphate (PLP) cofactor is connected to Lys40 through an internal aldimine bond and resides inside the α/β barrel domain.

As an incidental finding, the Alr_SP _structure contained additional electron density within the A monomer, at the end of helix 1 in the N-terminal α/β barrel domain. This planar density resembled a carboxylated aromatic ring, therefore a benzoic acid molecule, which fitted and refined well, was modeled into this region, even though the compound was not added to purification or crystallization conditions (topology and parameters obtained from the Hetero-compound Information Centre-Uppsala, HIC-UP [46]). It is situated some distance away from both the active site entryway and the dimer interface.

### Structural and biochemical comparison with closely related alanine racemases

**Figure 2:**
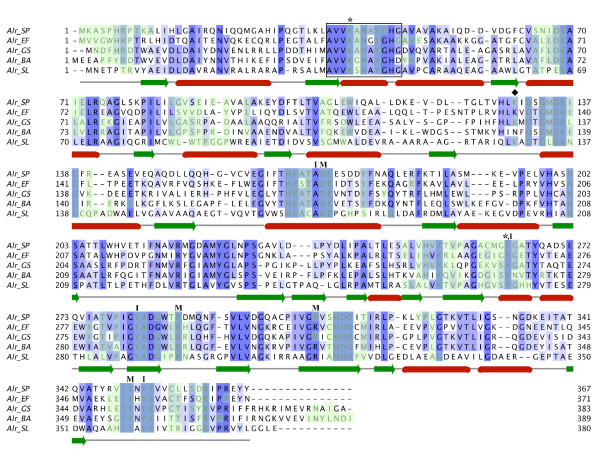
**Structure-based sequence alignment of the five solved alanine racemase structures from Gram-positive bacteria**. Structures are from *S. pneumoniae, G. stearothermophilus *[29], *E. faecalis *[38], *B. anthracis *[36] and *S. lavendulae *[33]. The black box encloses the conserved PLP binding site, the asterisks (*) mark the PLP-bound Lys residue and the catalytic Tyr residue, the diamond (♦) marks the location of the carbamylated Lys residue, and the residues constituting the entryway to the active site are marked with either **I **(inner layer) or **M **(middle layer). Residues that form intermonomer interfaces are highlighted in light green. The purple shading is proportional to the degree of sequence identity across the alignment.

As noted in our previous publication [21], Alr_SP _displays a high level of sequence similarity with other alanine racemases. The structure-based sequence alignment in Figure 2 demonstrates this similarity with alanine racemases from other Gram-positive bacteria: Alr_EF _(which has 52% sequence identity with Alr_SP_), Alr_GS _(46% identity), Alr_BA _(38% identity), and Alr_SL _(36% identity). Regions absolutely conserved across all of these enzymes include the characteristic PLP binding site motif near the N-terminus (AVVKANAYGHG), the two catalytic amino acid residues of the active center (Lys40, Tyr263'; throughout this paper, primed numbers denote residues from the second monomer) and the eight residues making up the entryway to the active site (inner layer: Tyr263', Tyr352, Tyr282', and Ala169; middle layer: Arg307', Ile350, Arg288', and Asp170).

**Figure 3:**
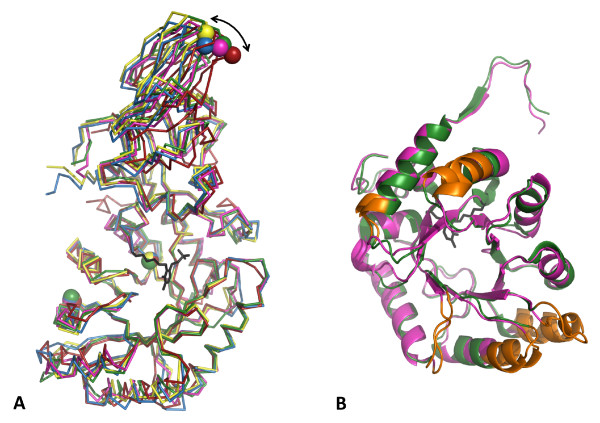
**Superposition of alanine racemase monomers from Gram-positive bacteria**. (A) Cα atom traces of alanine racemases from *G. stearothermophilus *(yellow) [29], *E. faecalis *(green) [38], *B. anthracis *(blue) [36], *S. lavendulae *(red) [33], and *S. pneumoniae *(pink). The superposed N-terminal α/β barrel domains are oriented on the bottom of the picture and the C-terminal β-strand domains on the top. Spheres represent the three structurally equivalent residues used to measure the hinge angle in each structure. The double-headed arrow indicates the variation between hinge angles. The PLP-bound Lys residue from Alr_SP _is shown in black. (B) Superposed ribbon representations of the N-terminal domains from *E. faecalis *(green) [38] and *S. pneumoniae *(pink), with the most divergent regions colored orange.

Superposition of the Cα atoms of monomer A from Alr_SP _with equivalent alanine racemase domains from other Gram-positive bacteria confirms the overall topological similarity between these structures (Figure 3A). There are minor conformational differences between these alanine racemases at the N- and C-termini and some loops in the α/β-barrel domain. Alr_SP _is similar in length to Alr_SL _and Alr_EF_; whereas Alr_GS _and Alr_BA _have 15 to 19 extra residues at the C-terminus that form an extra β-strand and helix/turn which contact the N-termini and the closest two helices of the α/β-barrel of each structure, and do not form part of the active site. The significance of these extra residues or lack thereof is unknown; future mutagenesis or domain-swap experiments may help to uncover their function.

Within each alanine racemase, the C- and N-terminal domains of each monomer are structurally distinct, and the hinge angle varies between the different enzymes [32,36], thereby preventing the optimal superposition of whole monomers. Overlaying the Cα atoms of Alr_SP _and alanine racemase structures from other Gram-positive bacteria results in average r.m.s. differences of 1.16-1.57 Å (Table 2), but when the N-terminal and C-terminal domains from Alr_SP _are superimposed separately, the C-terminal domain is shown to be more conserved (average r.m.s. differences of 0.49-1.24 Å), than the N-terminal domain (r.m.s. of 1.30-1.92 Å). Domain boundaries and residues used in these superpositions are listed in Table 3. The subset of residues found in the active site of Alr_SP _superpose very well with the equivalent residues of the other structures (r.m.s. of 0.36-0.67 Å). C-termini and active site residues superpose particularly well between Alr_SP _and Alr_GS_, and also between Alr_BA _and Alr_EF_. This structural similarity explains why Alr_GS _was such a successful molecular replacement model. Variability in the N-terminal domain is further illustrated by superposition of the N-terminal domains of Alr_SP _and its closest available homolog, Alr_EF_, which reveals significant deviations in Cα positions (≥1.8 Å) for five regions: residues 27-29, residues 53-58, residues 109-122, residues 150-156, and residues 192-196 (Figure 3B). The sequence in these regions is not highly conserved and they lie far from the active site. Superposition of the C-terminal domains from these structures shows no region with Cα differences greater than 1.7 Å. Overall, alanine racemase structures seem to tolerate significant alterations in the backbone of the α/β-barrel and β-domain and still retain almost identical active site residue locations.

**Table 2:** Average r.m.s. differences (Å) between the Cα atoms of Alr_SP _and alanine racemase structures from other Gram-positive bacteria

	PDB ID	Whole monomer	N-terminus	C-terminus	Active site
Alr_GS_	1SFT	1.23 (46%)	1.30 (41%)	0.57 (56%)	0.36 (66%)

Alr_SL_	1VFH	1.57 (38%)	1.92 (34%)	1.24 (41%)	0.67 (46%)

Alr_BA_	3HA1	1.29 (45%)	1.59 (41%)	0.49 (53%)	0.38 (65%)

Alr_EF_	3E5P	1.16 (53%)	1.48 (52%)	0.54 (56%)	0.46 (71%)

Numbers in parenthesis denote sequence identity with AlrSP, (%sequence identity = Nidentity/Naligned).

**Table 3:** Residues used in r.m.s. calculations

		**Alr**_**EF**_	**Alr**_**SP**_	**Alr**_**GS**_	**Alr**_**BA**_	**Alr**_**SL**_
**N-terminus**	monomer A	2-243	1-239	2-241	4-245	3-246

**C-terminus**	monomer A	244-371	240-367	242-388	246-389	247-378

**Active site**	monomer A	38-44	38-44	37-43	39-45	36-42
		
		62-66	61-65	61-65	63-67	60-64
		
		83-87	82-86	82-86	84-88	81-85
		
		100-104	101-105	101-105	103-107	100-104
		
		128-141	125-138	125-138	127-140	125-138
		
		164-172	160-168	161-169	163-171	163-171
		
		201-208	197-204	198-205	203-210	203-210
		
		219-226	215-222	216-223	221-228	221-228
		
		353-360	349-356	351-358	356-363	358-365
	
	monomer B	265-268	261-264	263-266	268-271	268-271
		
		311-316	307-312	309-314	314-319	315-320

The kinetic properties for Alr_SP _[21] are within the range of those previously observed for other bacterial alanine racemases (Table 4). The K_M _for L-alanine is 1.9 mM and V_max _for the racemization of L- to D-alanine is 84.8 U/mg, where one unit is defined as the amount of enzyme that catalyzes racemization of 1 μmol of substrate per minute. In the other direction, the K_M _for D-alanine is 2.1 mM and V_max _for the racemization of L- to D-alanine is 87.0 U/mg. However, the V_max _for the *S. pneumoniae *enzyme is more than one order of magnitude lower than that reported for the *G. stearothermophilus *and *E. faecalis *enzymes, even though the active site of Alr_SP _has high sequence and structural similarities with these alanine racemases. Differences of up to three orders of magnitude have been reported in this family despite very similar active sites.

**Table 4:** Kinetic parameters for the racemization of L- to D- and D- to L-alanine by alanine racemases from Gram-positive bacteria

	D to L direction			L to D direction		
**Enzyme [ref]**	**K**_***m***_**(mM)**	**V**_***max***_**(U/mg)**^**a**^	**k**_***cat***_**(min**^**-1**^**)**	**K**_***m***_**(mM)**	**V**_***max***_**(U/mg)**^**a**^	**k**_***cat***_**(min**^**-1**^**)**

**Alr**_**SP **_[21]	2.1^b^	87.0^b^	NR	1.9^b^	84.8^b^	NR

**Alr**_**GS **_[76]	2.7^c^	1400^c^	NR	4.3^c^	2550^c^	NR

**Alr**_**SL **_[77]	0.4^c^	NR	3800^c^	0.4^c^	NR	3300^c^

**Alr**_**BA **_[36]	2.8^b^	101^b^	NR	NR	NR	NR

**Alr**_**EF **_[78]	2.2^c^	1210^c^	~2340^c^	7.8^c^	3570^c^	~2340^c^



^a^One unit is defined as the amount of enzyme that catalyzes racemization of 1 μmol of substrate per minute.

^b^At 23°C. ^c^At 37°C. NR: not reported.

### Hinge angle

The hinge angle of the A monomer of Alr_SP_, formed by the Cα atoms of residues 99, 38 and 270 in the N-terminal α/β barrel domain and the C-terminal β-strand domain, is 132.3°. This is well within the range of hinge angles found between corresponding residues in the other Gram-positive alanine racemase structures (127.6° for Alr_BA_, 129.4° for Alr_GS_, 131.6° for Alr_EF_, and 138.2° for Alr_SL_). The difference in the degree of tilt between the C-terminal domains for the five structures can be seen in Figure 3A. Hydrogen bonding between the C- and N-terminal tails of opposite monomers was proposed by LeMagueres *et al. *to account for the distinct domain orientations of Alr_MT _and DadX_PA _[34]. Alanine racemase structures with extra residues at the N- and C-terminal tails, such as Alr_GS _and Alr_BA_, often form these hydrogen bonds, which are associated with smaller hinge angles (127.6° for Alr_BA_, 129.4° for Alr_GS_)[36].

Although the hinge angle clearly varies from species to species for this enzyme, the active sites superpose very well. Further, there is no correlation between hinge angle and Vmax (data not shown). On the other hand, there is some correlation between alanine racemase activity and bacterial doubling time. For example, the enzyme from the slow growing *M. tuberculosis *is very slow compared to the same enzyme from the rapid growing *M. smegmatis *species. It has previously been noted that only the dimeric form of the enzyme is active [47] and that many of the alanine racemase enzymes with the strongest monomer-dimer association have been found to be the most active [48]. A recent report has appeared looking at how enzyme activity in different alanine racemases relates to self-association affinity and this report confirms this assertion [49].

### Active site

**Figure 4:**
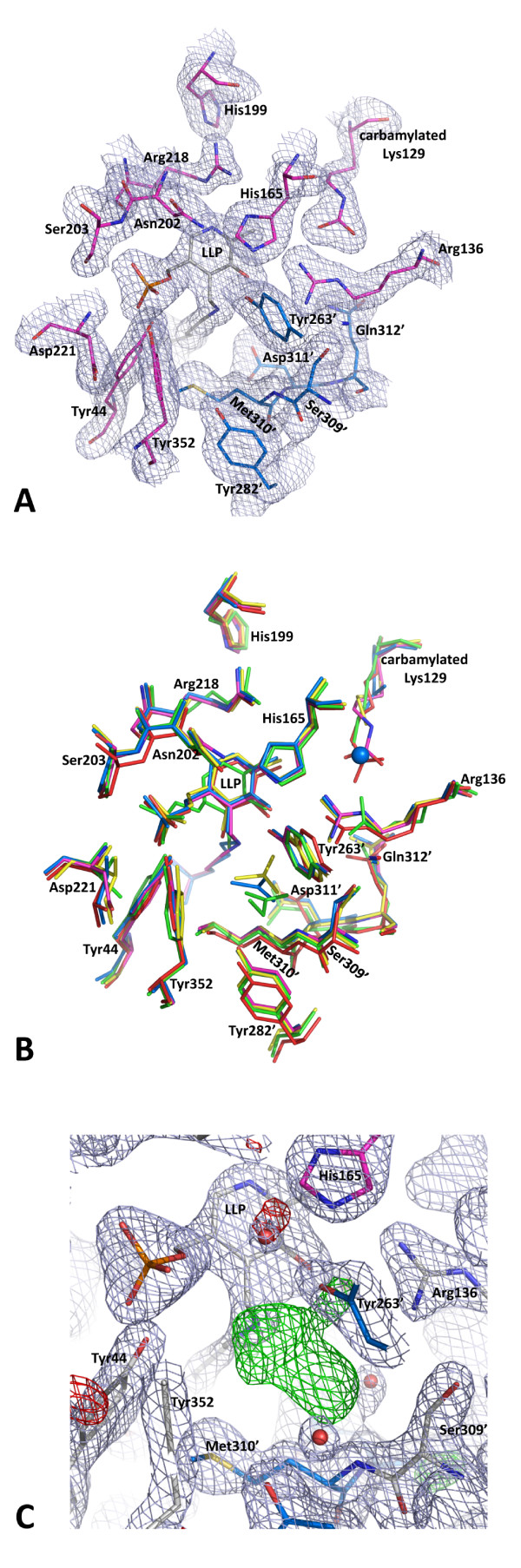
**Active site of alanine racemase from *S. pneumoniae***. (A) Electron density 2F_o_-F_c _map of the active site contoured at 1.5σ, excluding solvent. Residues from the first monomer are colored pink, residues from the second monomer are blue and are denoted with primed numbers. The PLP-bound Lys residue (LLP) is grey. (B) Superposition of the active site residues from Gram-positive alanine racemase structures with Alr_SP_; only *S. pneumoniae *residues are labeled. Residues pictured are from *G. stearothermophilus *(yellow) [29], *E. faecalis *(green) [38], *B. anthracis *(blue) [36], *S. lavendulae *(red) [33], and *S. pneumoniae *(pink). The chloride ion from the *B. anthracis *structure is depicted as a blue sphere. (C) Unmodeled electron density (green) found in the active site. 2F_o_-F_c _(light blue) and F_o_-F_c _(green and red) maps are contoured at 1.5 and 3.0 σ, respectively. Residues are colored and labeled as described for Figure 4A.

**Figure 5:**
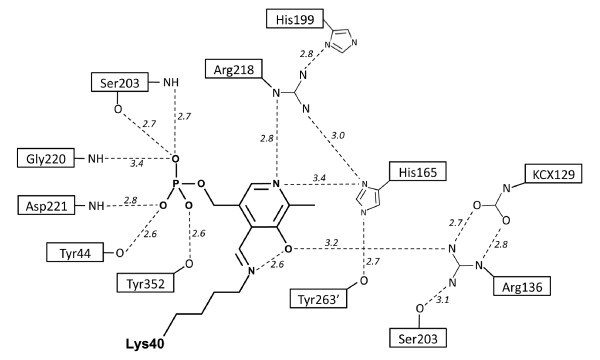
**Schematic diagram of polar interactions around PLP in the active site of alanine racemase from *S. pneumoniae***. For clarity, interactions with water molecules have not been included. Primed numbers denote residues from the second monomer. This figure was drawn after LeMagueres *et al. *[32].

The geometry and identities of the active site residues of Alr_SP _(Figure 4A) are very similar to that of other alanine racemases (Figure 4B). The main components of the Alr_SP _active site include the PLP cofactor covalently bound to Lys40 (forming an N'-pyridoxyl-lysine-5'-monophosphate or LLP residue), the catalytic base residue Tyr263' which lies at the beginning of helix 11 in the β-strand domain (contributed by the opposite monomer to that providing Lys40), and a hydrogen-bonded network of residues (Figure 5). Based on the reaction mechanism of alanine racemases proposed elsewhere [29-31,50], Alr_SP _operates by a two-base mechanism where the α-hydrogen of either D-alanine or L-alanine is abstracted and added by a different catalytic residue. Therefore, in the D- to L- direction, the reaction occurs with D-alanine binding to produce an external aldmine between PLP and D-alanine. Lys40 then abstracts the α-hydrogen to produce a carbanonic quinonoid intermediate. Next, Tyr263' adds a proton to the Cα of the intermediate from the opposite side to produce an external aldimine between PLP and what is now L-alanine. Subsequent transaldimination liberates L-alanine and regenerates the LLP form of the enzyme.

In the LLP moiety, the C4" atom of the PLP cofactor is linked to the NZ of Lys40 by a double bond in the *trans*- configuration, forming an internal aldimine as in other alanine racemase structures [29,31-33]. The PLP cofactor is further stabilized by hydrogen bonds with the side chains of six residues (Tyr44, Arg136, His165, Ser203, Arg218 and Tyr352) and main chains of three residues (Ser203, Gly220, Asp221; Figure 4A). The hydrogen-bonded network also includes residues His199 and Tyr263", and was first described in Alr_GS _[29]. All of these residues are strictly conserved across the Gram-positive structures, except for Asp221, which is replaced by an Ile in Alr_BA _and Alr_GS_, a Val in Alr_EF_, and a Leu in Alr_SL _[29,33].

We observed electron density consistent with a carbamylated lysine at the NZ terminus of Lys129, as seen in most other alanine racemase structures. Lys129 refined well as a carbamylated residue in this structure and is hydrogen bonded to the neighboring arginine residue. Shaw *et al. *[29] noted that this lysine residue is highly conserved, and later studies suggested that it helps to position the nearby arginine residue (Arg136 in Alr_SP_) which interacts with the substrate's carboxylate group through hydrogen-bonding [28,32]. Arg136 is further positioned in Alr_SP _by a hydrogen bond to Ser309. Sequences of alanine racemases that contain a lysine in position 129 almost always have an accompanying serine or cysteine residue in the equivalent of position 309 [36]. Recently, the Alr_BA _structure was found to contain an aspargine residue bound to a chloride ion at the equivalent position of Lys129, which appears to play the same role as the carbamylated Lys of positioning the active site arginine [36]. An alignment of alanine racemase sequences by Couñago *et al. *revealed that the presence of an aspargine residue can occur at the equivalent position of Lys129 in Alr_SP _and is likely to be indicative of an internal chloride within the active site in the place of a carbamylated lysine. Notably this change from Lys to Ser appears to always be accompanied by a threonine at the equivalent position of Ser309, even though the threonine does not directly interact with the chloride ion.

The environments on either side of the pyridine ring of PLP are quite different, as reported previously for Alr_GS _[29,33]. The side of the PLP that faces the dimer interface is polar in character, with many hydrophilic amino acid residues (including carbamylated Lys129, Arg136, His165 and Arg218), several water molecules and the hydrogen-bond network. The nonpolar side of PLP, in contact with the α/β barrel, contains several hydrophobic residues (Val38, Leu83, Leu85 and Phe163), no charged residues and no water molecules.

As observed in several other alanine racemase structures [29,32,34,36], we identified extra density in the active site of Alr_SP _adjacent to the PLP cofactor (Figure 4C). The position of this density corresponds to that of the acetate modeled in Alr_GS_. In other structures, this location has been reported to contain propionate, alanine phosphonate, and a putative substrate molecule in DadX_PA _[28-30,38]. Water molecules in the same location are found in the Alr_MT _and Alr_SL _structures. After unsuccessfully attempting to model a variety of small molecules into the extra density, including acetate, we left this region of the model empty.

### Active site entryway

**Figure 6:**
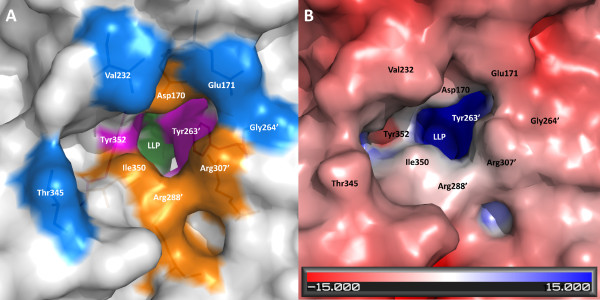
**Molecular surface representations of the entryway to the active site of alanine racemase from *S. pneumoniae***. (A) The surface of three layers of entryway residues: residues comprising the inner layer are pink (here, the constricting Tyr352 and Tyr263' residues can be seen), the middle layer residues are orange, and the outer layer residues are blue. The PLP cofactor is colored green. Primed numbers denote residues from the second monomer. (B) Surface of the entryway colored by electrostatic potential (same view as in A).

The entryway to the active site in Alr_SP _comprises the α/β barrel domain of one monomer and residues from the C-terminal domain of the other monomer, and is about 13 Å from the active site C4" atom of PLP. The entryway has a funnel-like shape, with its widest end towards the outside of the enzyme, narrowing as it approaches the PLP. The highly conserved residues comprising the entryway are distributed in layers beginning at the PLP site (Figures 6A and 6B): charged near the entrance, and mainly hydrophobic near the active site [33,34]. Mutagenesis has shown that these hydrophobic residues have an important role in controlling the substrate specificity of alanine racemase [51]. In Alr_SP_, the inner layer is made up of residues Tyr263', Tyr352, Tyr282', and Ala169. Residues Arg307', Ile350, Arg288', and Asp170 make up the middle layer. The residues composing the middle and the inner layers are strictly conserved between Alr_SP_, Alr_EF_, Alr_BA_, Alr_GS_, and Alr_SL_. An outer layer exists comprised of Thr345, Glu171, Val232 and Gly264', but these residues, which are able to interact with solvent directly, are not well conserved.

The Alr_SP _active site entryway includes the conserved pair of acidic residues Asp170 and Glu171. The equivalent residues in *E. coli*, Asp164 and Glu165, have been posited to play a role in substrate orientation [37]. Although the active sites of alanine racemases in general are moderate in size, it is difficult for inhibitors to access because of a constriction in the entryway corridor [34]. The smallest constriction in the entryway corridor of Alr_SP _is between Tyr263' and Tyr352 of the inner layer (Figure 6A), which provide an opening width of only about 2.6Å for an active site inhibitor to pass through (the distance between the closest atoms of these two side chains with the van der Waals radius for each atom subtracted). As a result, the substrate entryway itself has been proposed as an alternative target for inhibitor development [32,34]. Wang *et al. *[52] have proposed this idea previously for another enzyme, histone deacetylase-like protein.

### Dimer interface

Dimerization is essential for the catalytic activity of alanine racemase [47]. Both monomers contribute to the overall composition of the active site, the alanine entryway, and the binding pocket. Within the Alr_SP _dimer interface there are 33 hydrogen bonds and 10 salt bridges (Table 5). There are no disulfide or covalent bonds across the interface. 91 residues from each monomer are involved in intermonomer interactions. The buried surface areas of the A and B monomers are 3035 and 3020 Å^2^, respectively; both values are 19% of the total surface area of each monomer. The interface surface area is similar to that seen in the closely related Alr_EF _and Alr_GS _(Table 5). 30% of the interface residues in Alr_SP _are polar, 47% are non-polar, and 22% are charged.

**Table 5:** Intermonomer interactions for alanine racemases from Gram-positive bacteria calculated using PISA [69] and PROTORP [70]

	Buried surface area (Å^2^)		H-bonds	Salt link	# residues		% interface residues		
				
	Monomer A	Monomer B			A	B	polar	non-polar	charged
Alr_SP_	3035	3020	33	10	91	91	30	47	22

Alr_GS_	3058	3109	43	17	89	88	27	43	30

Alr_SL_	2800	2799	26	6	87	87	39	43	18

Alr_BA_	3529	3529	45	10	93	95	28	44	27

Alr_EF_	2936	2936	38	12	86	86	32	41	28

Disruption of the dimer interface could be used to inhibit alanine racemase activity, and has been successfully used on drug targets in HIV and HCV [53-55], and on caspases [56]. Inspection of the residues that participate in the dimer interface of Alr_SP _on a structure-based sequence alignment (Figure 2) makes it apparent that that many of these residues are highly conserved, and also participate in substrate guidance (such as middle and inner entryway residues Tyr282', Tyr352, Arg307', Ile350, Arg288', Asp170) or catalysis (e.g. Lys40, Tyr263').

### Pentagonal water molecules in the active site

**Figure 7:**
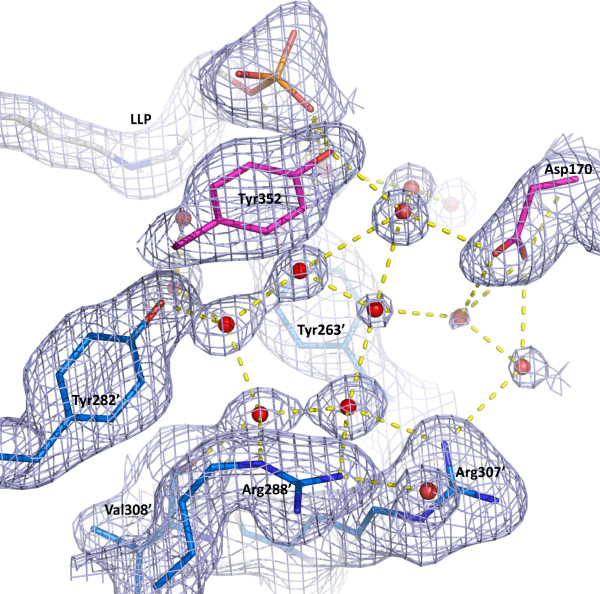
**Pentagonal ring waters near the substrate binding site in alanine racemase from *S. pneumoniae***. The electron density 2F_o_-F_c _map is contoured at 0.8σ. Residues are shown as sticks, red spheres represent water molecules, and dashed yellow lines represent hydrogen bonds. Residues from the first monomer are colored pink, residues from the second monomer are blue and are denoted with primed numbers. The PLP-bound Lys residue (LLP) is grey. For simplicity, only some of the residues are shown.

A cluster of hydrogen-bonded water molecules forms an ordered pentagonal ring and some adjacent partial rings in the active site entryways of both monomers of Alr_SP _(Figure 7). The pentagonal ring waters are located adjacent to the substrate binding site and between residues Tyr263" and Tyr282". They are positioned at the interface of monomer A and B and appear to be involved in the dimer interface, making direct or indirect hydrogen bonds with interface residues (Asp170, Tyr263', Tyr282', Tyr288', Arg307', Tyr352). The distance between the water oxygen atoms that form each side of the pentagon is about 2.7 Å. The pentagonal ring is hydrogen-bonded directly to the protein at five atoms (Tyr282' OH, Arg307' NH1, and Arg288' NH2 and NE from the entryway inner and middle layers, and Val308' O) and makes hydrogen bonds with other waters both deeper in the active site and at the outer region of the entryway.

The hydrogen bond network we have identified could be facilitating substrate movement or proton transfer into the active site. Analysis of conserved water sites in Alr_GS _has been reported previously and the authors postulated that these sites could be involved in proton transfer or solvent shift into the active site [57]. In the high resolution structure of the protein crambin, Teeter reported pentagonal rings of water molecules which were felt to have a role in stabilizing protein structure or in catalysis [58]. Pentagonal ring waters have not yet been reported for other alanine racemases, but depending on which monomer is observed, the Alr_BA _and Alr_GS _structures have 3 to 4 of the 5 pentagonal waters present in Alr_SP_, the Alr_SL _structure has 2 of the 5 pentagonal waters, and the Alr_EF _structure does not have any pentagonal ring waters, although this could be due to limits in structural resolution.

### Alanine racemase as a target for drug design

In this section we review some of the challenges encountered in developing inhibitors for alanine racemases as a family and we explain the contribution of the *S. pneumoniae *structure to this process. Finally we offer our assessment of the most useful approaches to alanine racemase inhibitor development.

Challenges involved in designing inhibitors for alanine racemase are easy to identify. To begin with, there have been few reports to date of alanine racemase inhibitors with any true specificity. Incorporating features of the active site in drug design has been challenging because the structure of the active site is thought to have limited accessibility. Further, several inhibitors have been found to cross react with human enzymes that contain PLP. Even so, our analysis of alanine racemase structures has allowed us to identify key features that could be incorporated into the inhibitor development process. Since these key features are also present in the *S. pneumoniae *enzyme structure, it confirms that these features are not artifacts or incidental findings but conserved features that can be targeted in the development of a class of inhibitors specific to bacterial alanine racemases. Therefore the structure of the *S. pneumoniae *enzyme is valuable to racemase drug design efforts.

In addition, one new feature relevant to the traditional drug design approach of blocking the active site that we report here for Alr_SP _is the pentagonal water network within the active site. Several of these waters are conserved in other alanine racemase species. That being the case, the conserved waters could be incorporated within an *in silico *pharmacophore as a polar site capable of receiving or donating a hydrogen bond depending on its protonation state. Unfortunately, to date testing of compounds identified from *in silico *screening has not resulted in the identification of strong inhibitors.

The earliest drug development work on alanine racemase was carried out in the absence of a crystal structure and resulted in the development of a cycloserine, a small, covalent inhibitor of alanine racemase and other PLP-containing enzymes [59] that lacks any specific interactions with elements in the active site. More recent *in silico *drug design work carried out using the structure of alanine racemase has defined a pharmacophore situated within the active site near the alanine racemase acetate binding site, a site reported consistently within alanine racemase structures [60]. However, analysis of the narrow entryway to the active site PLP suggests that access to the proposed interior binding pockets of the enzyme is likely to be limited, especially for larger compounds [32,34]. To be an effective drug target it is important the active site be accessible, therefore standard structure-aided inhibitor design approaches are limited for alanine racemase.

Since, as illustrated by the Alr_SP _structure, entryway to the interior binding pocket of alanine racemase is very tight, the drug design problem for alanine racemase resembles a "ship in a bottle" (SIAB) dilemma. Once inside the interior pocket, the compounds proposed to bind to the active site would fit well but these compounds may only make it to the interior with difficulty [32,34,36]. This view is of course an oversimplification, as the entryway is likely to 'breathe' and adjust, and there is a monomer-dimer equilibrium for alanine racemase that would affect the geometry and accessibility of internal active site cavities. However, the restricted access and size of the alanine racemase active site is one reason it has not been targeted by major pharmaceutical companies in the recent past (Bussiere, Dirk; personal communication).

If a drug design project involving an enzyme with a SIAB active site is to be successful, there are four obvious approaches to inhibitor development: high throughput screening (HTS), blocking the opening, interfering with active site assembly, or developing drugs that enter in one shape and adopt a new conformation after binding, thus trapping them in the active site.

HTS would bypass any of the complexities associated with active site access and would provide a set of compounds that inhibit the enzyme by any and all means, to be deconvoluted later. Given that the active site features we describe for the *S. pneumoniae *enzyme are highly conserved in the bacterial structures reported to date, the alanine racemase inhibitors identified by HTS would likely be broad-spectrum in their action. But a broad spectrum of activity should not be viewed in a negative light, as almost all major classes of antibiotics developed to date are broad spectrum. This includes beta-lactams like penicillin and cephalosporins, fluoroquinolones, tetracyclines, even macrolides. In fact the only specificity among anti-bacterial classes currently in use would be that some target preferentially Gram-positives, Gram-negatives, mycobacteria or anaerobes.

Blocking the opening would involve the design of compounds that interact with residues in the entryway and that extend toward the PLP moiety, but that might not reach the interior binding pocket. In our previous work on the alanine racemase from *P. aeruginosa, M. tuberculosis *and *B. anthracis*, we described a highly conserved and layered entryway to the active site that contains both hydrophobic and polar features. The hydrophobic regions are bound by three tyrosines and an alanine in the inner layer of entryway, while the polar areas include two arginines and one aspartate located in the middle layer. These highly conserved features are present in the *S. pneumoniae *structure and all alanine racemase structures reported to date. An entryway of this type has not been described in human PLP-containing enzymes. For example, human serine racemase, which like alanine racemase reacts with D-cycloserine, belongs to the Fold type II family of PLP-containing enzymes and would be not be expected to cross-react with any specific alanine racemase inhibitors [61]. The alanine racemase topology is termed Fold type III and is unique among PLP-containing enzymes. It seems likely, therefore, that designing inhibitors that interact with conserved motifs found in the entryway could represent a potential source of specificity in the drug design process.

Interfering with active site assembly would, in the case of alanine racemase, require compounds that inhibit dimer formation, none of which have been reported for alanine racemase to date. However, dimer inhibitors have been reported in other systems such as HIV protease [53-55]. Finally, a compound that could enter the active site of alanine racemase then undergo a conformational switch rendering the enzyme inactive would make an effective inhibitor, but this type of inhibitor has not yet been reported for this class of enzyme.

## Conclusions

Alanine racemase is a promising target for antibacterial drugs because it is both essential in bacteria and absent in humans. We report the high-resolution crystal structure of alanine racemase from *S. pneumoniae*. Overall, the structure shares the conserved active site and topology found across all alanine racemases. Known alanine racemase inhibitors such as D-cycloserine, alanine phosphonate, and other substrate analogues are not specific, acting on other PLP-containing enzymes such as transaminases, also found in humans [59,62]. In order to be clinically relevant, new inhibitors of alanine racemase with more specificity need to be developed. This structure is an essential starting point for the design of more specific inhibitors of alanine racemase in *S. pneumoniae*. Our investigations have identified three potential areas in the Alr_SP _structure that could be targeted in a structure-based inhibitor design: the active site, the residues forming the dimer interface, and the active site entryway in particular, since designing a 'plug' to fit the funnel shape of this feature is intuitively attractive.

## Methods

### Protein expression, purification and crystallization

The expression, purification and crystallization of Alr_SP _have been described previously [21]. Briefly, the gene encoding Alr_SP _was cloned into pET17 (Novagen) and the resulting vector transformed into *E. coli *BL21 (DE3) pLysS cells (Novagen). Overexpression of Alr_SP _was induced in a culture of these cells, which were then lysed to extract the protein. The recombinant Alr_SP _was purified using ammonium sulfate precipitation, anion-exchange chromatography, hydrophobic interaction chromatography, and finally, size-exclusion chromatography. Crystals of Alr_SP _were grown at 4°C in 1.2 M Na Citrate, 0.1 M MES, pH 7.2, and 10% glycerol (protein concentration 23 mg/ml, drop size 4 μl + 4 μl) using the sitting drop vapor diffusion method, then flash-frozen in liquid N_2 _for data collection. No additional cryoprotectant was required.

### Data collection and processing

Diffraction data were collected to 2.0 Å resolution at 100 K using a Rigaku FR-E generator and an HTC detector at 45 kV and 45 mA with Cu Kα radiation at Rigaku MSC (The Woodlands, TX). The crystals belonged to the space group P3_1_21 with the unit cell parameters a = b = 119.97 Å, c = 118.10 Å, α = β = 90° and γ = 120°. The data were processed and merged using the HKL package version 1.96.6 [63]. Data collection and processing statistics are listed in Table 1.

### Structure determination and refinement

The structure of Alr_SP _was solved by molecular replacement using CNS version 1.1 [42]. Alr_GS _(PDB ID 1SFT) [29] without the PLP cofactor was used as a search model, and two monomers per asymmetric unit were assumed, as suggested by a Matthews coefficient [64] of 3.0 with a solvent content of 59.0%. Cross-rotation and translation searches were completed and the best solution was used as an initial model for model building. After rigid body refinement in CNS, ARP/wARP version 6.1 [65] was used to trace the initial protein model and build side chains. Further refinement was carried out using simulated annealing and conjugation gradient minimization. When 97% of residues were built, the co-factor PLP and the carbamylated lysine were placed, and positional and B-factor refinements were continued resulting in an R and R_free _of 31.9 and 33.9%, respectively. Water molecules were added using the water-picking script in CNS, and further cycles of positional and B_iso _refinements brought the R and R_free _to 20.7 and 25.7%, respectively. Since previous alanine racemase structures have shown indications of subdomain movement, we tried TLS refinement [43]. We used the TLS motion determination server [66,67] to produce modified PDB files and TLS input files for the structure partitioned into either one, five or twenty TLS groups, then further refined these models in Refmac5 version 5.5.0109 [44]. All models resulted in similar improvements in R and R_free _so we used the simplest option, which treated all protein atoms found in the asymmetric unit as a single rigid body (one TLS group). PLP and Lys40 were replaced with an LLP residue (PLP covalently bound to lysine), and TLS refinement was completed using Refmac5. The final model has an R and R_free _of 16.8 and 20.0%, respectively. Refinement statistics are listed in Table 1. Structure factors and final atomic coordinates for AlrSP have been deposited in the Protein Databank (PDB ID: 3S46). B-factor values and correlation coefficients were calculated using the programs Baverage and Overlapmap from the CCP4 suite [44].

### Structural and sequence comparisons

The multiple structure-based sequence alignment and structural superpositions of Alr_SP _with closely related structures were performed using the protein structure comparison service (SSM) at the European Bioinformatics Institute (http://www.ebi.ac.uk/msd-srv/ssm) [68]. The PDB IDs of the structures used were: 1SFT (Alr_GS_), 1VFH (Alr_SL_), 3HA1 (Alr_BA_), and 3E5P (Alr_EF_). Table 3 lists the residues from these structures used in the superpositions. Intermonomer interactions were analysed using the Protein Interfaces, Surfaces and Assemblies service (PISA) at the European Bioinformatics Institute (http://www.ebi.ac.uk/msd-srv/prot_int/pistart.html) [69], and the Protein-Protein interface analysis server (PROTORP) Server (http://www.bioinformatics.sussex.ac.uk/protorp/ index.html) [70].

## Figure preparation

Representations of molecules were prepared using the programs PyMOL [71] and BKChem (http://bkchem.zirael.org/index.html). The sequence alignment was visualized using Jalview [72]. The electrostatic potential of the Alr_SP _surface was calculated using the Adaptive Poisson-Boltzmann Solver (APBS) [73] through PyMOL. Default configurations were used for calculations. PQR files for use with APBS were generated using the PDB 2PQR Server (http://kryptonite.nbcr.net/pdb2pqr/) [74] and the Dundee PRODRG2 Server (http://davapc1.bioch.dundee.ac.uk/prodrg/) [75].

## Authors' contributions

HI performed research, helped draft the manuscript, analyzed results and prepared figures. MS helped to refine the structure and draft the manuscript, analyzed results and prepared figures. US and MD performed research and critically appraised the manuscript. KK designed research, supervised the work, organized financial support, and critically appraised the manuscript. All authors read and approved the final manuscript.
